# Milk Fat Globule-EGF Factor 8 Alleviates Pancreatic Fibrosis by Inhibiting ER Stress-Induced Chaperone-Mediated Autophagy in Mice

**DOI:** 10.3389/fphar.2021.707259

**Published:** 2021-08-05

**Authors:** Yifan Ren, Qing Cui, Jia Zhang, Wuming Liu, Meng Xu, Yi Lv, Zheng Wu, Yuanyuan Zhang, Rongqian Wu

**Affiliations:** ^1^ National Local Joint Engineering Research Center for Precision Surgery and Regenerative Medicine, Shaanxi Provincial Center for Regenerative Medicine and Surgical Engineering, First Affiliated Hospital of Xi’an Jiaotong University, Xi’an, China; ^2^ Department of General Surgery, The Second Affiliated Hospital of Xi’an Jiaotong University, Xi’an, China; ^3^ Department of Cardiology, Xi’an Central Hospital, Xi’an, China; ^4^ Department of Hepatobiliary Surgery, First Affiliated Hospital of Xi’an Jiaotong University, Xi’an, China; ^5^ Department of Department of Pediatrics, First Affiliated Hospital of Xi’an Jiaotong University, Xi’an, China

**Keywords:** chronic pancreatitis, MFG-E8, fibrosis, pancreatic stellate cell, chaperone-mediated autophagy, LAMP2A

## Abstract

Pancreatic fibrosis is an important pathophysiological feature of chronic pancreatitis (CP). Our recent study has shown that milk fat globule-EGF factor 8 (MFG-E8) is beneficial in acute pancreatitis. However, its role in CP remained unknown. To study this, CP was induced in male adult *Mfge8*-knockout (*Mfge8*-KO) mice and wild type (WT) mice by six intraperitoneal injections of cerulein (50 μg/kg/body weight) twice a week for 10 weeks. The results showed that knockout of *mfge8* gene aggravated pancreatic fibrosis after repeated cerulein injection. In WT mice, pancreatic levels of MFG-E8 were reduced after induction of CP and administration of recombinant MFG-E8 alleviated cerulein-induced pancreatic fibrosis. The protective effect of MFG-E8 in CP was associated with reduced autophagy and oxidative stress. In human pancreatic stellate cells (PSCs), MFG-E8 inhibited TGF-β1-induced ER stress and autophagy. MFG-E8 downregulated the expression of lysosomal associated membrane protein 2A (LAMP2A), a key factor in ER stress-induced chaperone-mediated autophagy (CMA). QX77, an activator of CMA, eliminated the effects of MFG-E8 on TGF-β1-induced PSC activation. In conclusion, MFG-E8 appears to mitigate pancreatic fibrosis *via* inhibiting ER stress-induced chaperone-mediated autophagy. Recombinant MFG-E8 may be developed as a novel treatment for pancreatic fibrosis in CP.

## Introduction

Pancreatic fibrosis is an important pathophysiological feature of chronic pancreatitis (CP). Its management remains a serious clinical challenge (Kleeff et al., 2017; Gardner et al., 2020). Activation of the pancreatic stellate cell (PSC) is a key step in the development of pancreatic fibrosis (Ren et al., 2020). In normal pancreas, PSCs surround acinar cells in a resting state. When the pancreas is challenged by inflammation or mechanical stimulation, PSCs’ phenotype changes from a resting to an active state (Bynigeri et al., 2017). The production of α-SMA is a hallmark of PSC activation, and is accompanied by the production of large amounts of collagen I and III (Xue et al., 2018), which together form the pathological process of pancreatic fibrosis in CP. However, the molecular mechanism of PSC activation during the development of CP is still obscure.

Autophagy is involved in the activation of PSCs (Endo et al., 2017; Ren et al., 2020). Suppressing autophagy has been shown to inhibit the activation of PSCs and reduce the development of pancreatic fibrosis (Xue et al., 2019). Chaperone-mediated autophagy (CMA) is a process in which lysosomal degradation occurs when cytoplasmic proteins with special modules are recognized by molecular chaperones and bind to lysosomal-associated membrane protein 2A (LAMP2A) (Kaushik and Cuervo, 2018). LAMP2A, a special receptor on the lysosomal membrane, is believed to be a key regulator of CMA (Pajares et al., 2018). Endoplasmic reticulum (ER) stress is an important trigger of CMA (Li et al., 2017). Our previous study has shown that activation of PSCs is associated with oxidative and ER stress (Ren et al., 2020). However, the specific role of CMA in pancreatic fibrosis during CP remains largely unknown.

Milk fat globule epidermal growth factor (EGF) factor 8 (MFG-E8), also known as lactadherin, is a lipophilic glycoprotein. It is expressed and secreted by a variety of cells and tissues including the pancreas (D’Haese et al., 2013). MFG-E8 contains an RGD motif and can interact with integrins (Franchi et al., 2011; Aziz et al., 2015). Through binding to integrin receptors, MFG-E8 exhibits versatile functions and is involved in a variety of cellular processes, such as maintenance and repair of intestinal epithelial cells, angiogenesis, and clearance of apoptotic cells (Kranich et al., 2010; Deng et al., 2017; Gao et al., 2018). Previous studies have indicated that MFG-E8 inhibited the activation of fibroblasts induced by TGF-β1, and recombinant MFG-E8 alleviated the development of fibrosis in the skin, heart, kidney and liver in mice (An et al., 2017; Fujiwara et al., 2019; Shi et al., 2020; Wang et al., 2020). Our recent study has shown that MFG-E8 restores mitochondrial function in acute pancreatitis (Ren et al., 2021). However, whether MFG-E8 plays any role in the development of pancreatic fibrosis has not been studied. The main purpose of the current study is to investigate the role of MFG-E8 in ER stress, CMA, PSC activation and pancreatic fibrosis in CP.

## Materials and Methods

### Experimental Animal

C57BL/6J adult mice were purchased from Animal Experimental Center of Xi’an Jiaotong University Health Science Center, and *Mfge8*-knockout (*Mfge8*-KO) mice were purchased from Nanfang Biotech Technology Co., Ltd (Shanghai, China). *Mfge8*-KO mice were obtained by knocking out 2–6 exons of *mfge8* gene using CRISPR/Cas9 gene editing technology. All experimental animals are housed in a temperature-controlled room on a 12-h light/dark cycle with *ad libitum* access to food and water. The mice were fasted for 12 h before collecting samples. The study protocol was approved by the Institutional Animal Care and Use Committee of the Ethics Committee of Xi’an Jiaotong University Health Science Center.

### Mouse Model of Chronic Pancreatitis and Administration of MFG-E8

Chronic pancreatitis was induced in male adult mice by six intraperitoneal injections of cerulein (50 μg/kg/body weight, C6660, Solarbio, Beijing, China) twice a week for 10 weeks, as described by Sendler M. et al. (Sendler et al., 2015) and used by us recently (Ren et al., 2020). During the last 5 weeks, 1 hour after cerulein injection, normal saline (vehicle) or 20 μg/kg recombinant murine MFG-E8 (RD System, Inc. Minnesota, United States) was administered through intraperitoneal injection. The animals were sacrificed 2 days after the last injection of cerulein. The doses of MFG-E8 used in this study were chosen on the basis of our previous publications in acute pancreatitis (Ren et al., 2021). Blood and tissue samples were collected.

### Cell Culture and Treatment

Human pancreatic stellate cells (PSCs) were purchased from FengHui Biotechnology (FHHUM-CELL-0124, China) and cultured in Ham’s F-12K medium (PM150910C, Procell, Wuhan, China) with 20% fetal bovine serum (164210-100, Procell, Wuhan, China) in a humidified incubator at 37°C with 5% CO_2_. HPSCs (1X10^6^/well) were planted into 6-well plates for 24 h. The cells appeared to be in quiescent state. Then, the cells were treated with 5 ng/mL TGF-β1 (5154LC, Cell Signaling Technology, United States) with 10 ng/ml or 20 ng/mL recombinant human MFG-E8 or equal volume of PBS for 24 h. In additional groups of cells, QX77 (5 ng/ml, S6797, SELLEK, United States), a LAMP2A-specific activator (Zhang et al., 2017), was added simultaneously with 20 ng/mL MFG-E8 in TGF-β1-treated HPSCs. 24 h later, the cells were collected for various measurements.

### Histologic Evaluation

Pancreatic tissue sections were stained with H&E. The pathological staining scoring system introduced by Schmidt et al. (Schmidt et al., 1992) was used to evaluate the pancreatic tissue damage.

### Immunohistochemical Staining

Immunohistochemical staining was performed as we described before (Bi et al., 2020). Paraffin sections of mouse pancreatic tissue were prepared, and Sirius red and Masson-Goldner staining were used to indicate the degree of tissue fibrosis. α-smooth muscle actin (α-SMA) staining (A5228, mouse monoclonal clone, Sigma-Aldrich, United States) and Collagen I (ab34710, rabbit polyclonal, Abcam, United States) staining were used to mark the deposition of extracellular matrix. MPO (ab45977, rabbit polyclonal, Abcam, United States) was used to mark the infiltration of neutrophils. Gr1 (LY6G) (ab25377, antibody, Abcam, United States), CD11b (ab216445, rabbit polyclonal, Abcam, United States) and F4/80 (ab240946, rabbit polyclonal, Abcam, United States) were used to demonstrate macrophage infiltration, LC3B (ab48394, rabbit polyclonal, Abcam, United States) were used to demonstrate autophagy levels. The immunohistochemical staining was photographed with a light microscope. Three fields were randomly selected for each image, and the images were quantitatively and statistically analyzed with ImageJ Pro Plus 6.0 software.

### Immunofluorescence Staining

The expression of Collagen I, α-SMA and DHE were assessed by immunofluorescence staining as described by us previous (Ren et al., 2020). Quantitative determination of fluorescence intensity was perfumed by Image Pro Plus 6.0 software.

### Enzyme-Linked Immunosorbent Assay

The mouse IL-6 ELISA kit (SEA079Mu, Cloud-Clone Corp USCN Life Science, Wuhan, CN), tumor necrosis factor-α (TNF-α) ELISA kit (SEA133Mu, Cloud-Clone Corp USCN Life Science, Wuhan, CN), IL-10 ELISA kit (SEA056Mu, Cloud-Clone Corp USCN Life Science, Wuhan, CN) and MFG-E8 ELISA kit (SEB286Mu, Cloud-Clone Corp USCN Life Science, Wuhan, CN) were used for the detection of IL-6, TNF-α, IL-10 and MFG-E8 according to the manufacturer’s instructions.

### Detection of SOD, FRAP GSH and MDA Levels

Pancreatic tissue and HPSCs homogenate was obtained and superoxide dismutase (SOD), total antioxidant capacity assay kit with FRAP method (FRAP value), glutathione (GSH) and malonaldehyde (MDA) were measured as we described before (Ren et al., 2019a; Ren et al., 2019b; Ren et al., 2020).

### Western Blot Analysis

Pancreatic tissues were lysed in cold RIPA (P0013B, Beyotime, Beijing, China). The protein concentration was evaluated with the BCA Protein Assay Kit (P0012S, Beyotime, Beijing, China). After gel electrophoresis, the protein was transferred to PVDF membrane and incubation in blocking solution (3% BSA or 5% skimmed milk) at room temperature. Then the membranes were incubated overnight at 4°C with the primary antibodies (Supplementary Table S1). Primary antibodies were diluted in Primary Antibody Dilution Buffer for Western Blot (P0256, Beyotime, Beijing, China). Membranes were washed and then incubated with specific HRP‐conjugated secondary antibodies (Supplementary Table S1) for 1.5 h at room temperature. Bands were developed using Digital gel image analysis system (Bio‐Rad, California, United States) and quantitative of protein level were calculated by ImageJ2x software. Information about the antibodies used in this study are listed in Supplementary Table S1.

### Statistical Analysis

All measurement data are expressed as the mean ± standard error (SEM). The *t*-test or one-way ANOVA with the Tukey-Kramer test was used to analyze the differences between groups. All analyses were conducted with data statistics software GraphPad Prism version 8.0 (GraphPad Software, Inc., San Diego, CA, United States). *p* < 0.05 represented a significant difference.

## Results

### MFG-E8 Deficiency Exaggerates Repeated Cerulein Injection-Induced CP *in Vivo*

**FIGURE 1:**
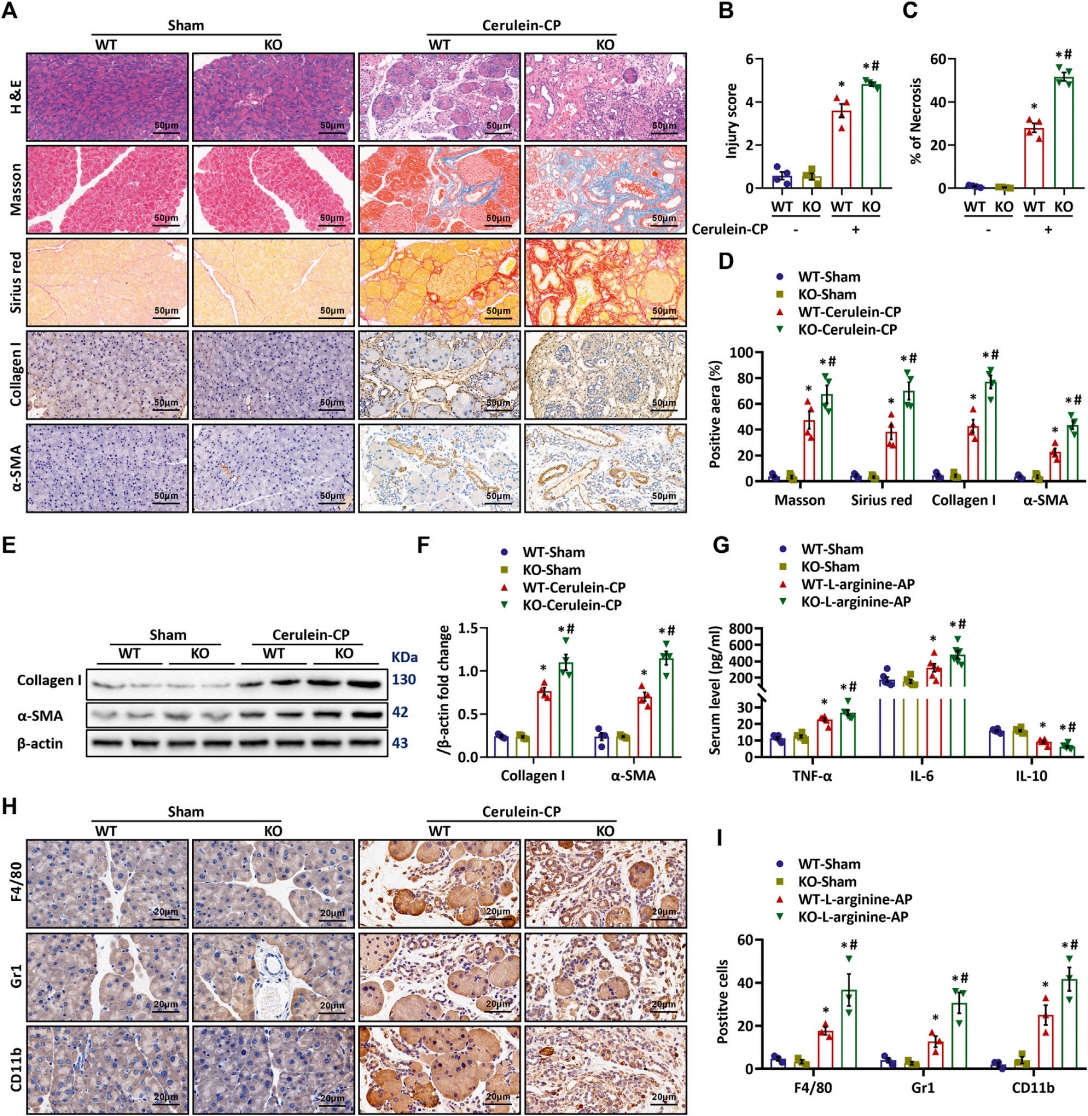
**MFG-E8 Deficiency Potentiates Cerulein-Induced CP *in Vivo*.** Cerulein-CP was induced by six IP injections of cerulein (50 μg/kg/body weight) twice a week for 10 weeks. During the last 5 weeks, 1 h after cerulein injection, normal saline (vehicle) or 20 μg/kg MFG-E8 was administered through intraperitoneal injection. The control group received the same frequency and time of intraperitoneal injection of normal saline (Sham). The animals were sacrificed at 2 days after the last injection of cerulein or normal saline. Blood and tissue samples were collected. **(A)** Representative photos of H&E, Sirius red, Masson, Collagen I and α-SMA staining; **(B)** Quantitative analysis of H&E staining (one-way ANOVA with the Tukey-Kramer test); **(C)** Percentages of necrotic areas (one-way ANOVA with the Tukey-Kramer test); **(D)** Quantitative analysis of Sirius red Masson, Collagen I and α-SMA staining (one-way ANOVA with the Tukey-Kramer test); **(E,F)** Western blot analysis of the expression of α-SMA and collagen I in the pancreas (one-way ANOVA with the Tukey-Kramer test); **(G)** Serum levels of IL-6, IL-10 and TNF-α (one-way ANOVA with the Tukey-Kramer test); **(H,I)** Representative photos and quantitative analysis of F4/80, Gr1 and CD11b staining (one-way ANOVA with the Tukey-Kramer test). *n* = 4–6, mean ± SEM; ∗ *p* < 0.05 versus Sham group; #*p* < 0.05 versus Vehicle group. CP, chronic pancreatitis; H&E, hematoxylin and eosin; α-SMA, alpha-smooth muscle actin; WT, wild type; KO, knock out.

To evaluate the pathophysiological role of MFG-E8 in CP, we induced CP in *Mfge8*-KO mice (The efficacy of MFG-E8 knockout was confirmed by Western blotting, Supplementary Figure S1) by repeated intraperitoneal injection of cerulein. H&E staining showed that MFG-E8 deficiency did not result in any significant changes in pancreatic histomorphology in sham mice (Figure 1A). However, repeated cerulein injection caused much more severe pancreatic damage in *Mfge8*-KO mice than their WT littermates (Figures 1A,B). *Mfge8*-KO mice also had larger area of necrosis than WT mice after repeated cerulein injection (Figures 1A,C). Masson and Sirius red staining showed more severe fibrosis in the pancreatic tissue of *Mfge8*-KO mice than that of WT mice (Figures 1A,D). Consistently, α-SMA and Collagen I staining showed that repeated cerulein injection caused extracellular matrix deposition in the interstitial space of *Mfge8*-KO mice than that of WT mice (Figures 1A,D). These findings were confirmed by western blot analysis of α-SMA and Collagen I protein expression in the pancreatic tissues (Figures 1E,F). MFG-E8 Deficiency also potentiated inflammatory responses in CP. As shown in Figure 1G, serum proinflammatory cytokines TNF-α and IL-6 were further increased, while anti-inflammatory cytokine IL-10 was further decreased in *Mfge8*-KO mice than WT mice after repeated cerulein injection. Inflammatory cell infiltration in the pancreas was measured by F4/80, CD11b, and Gr1 staining. As shown in Figures 1H,I, repeated cerulein injection also resulted in more inflammatory cell infiltration in the pancreas of *Mfge8*-KO mice than that of WT mice.

**GRAPHICAL ABSTRACT:**
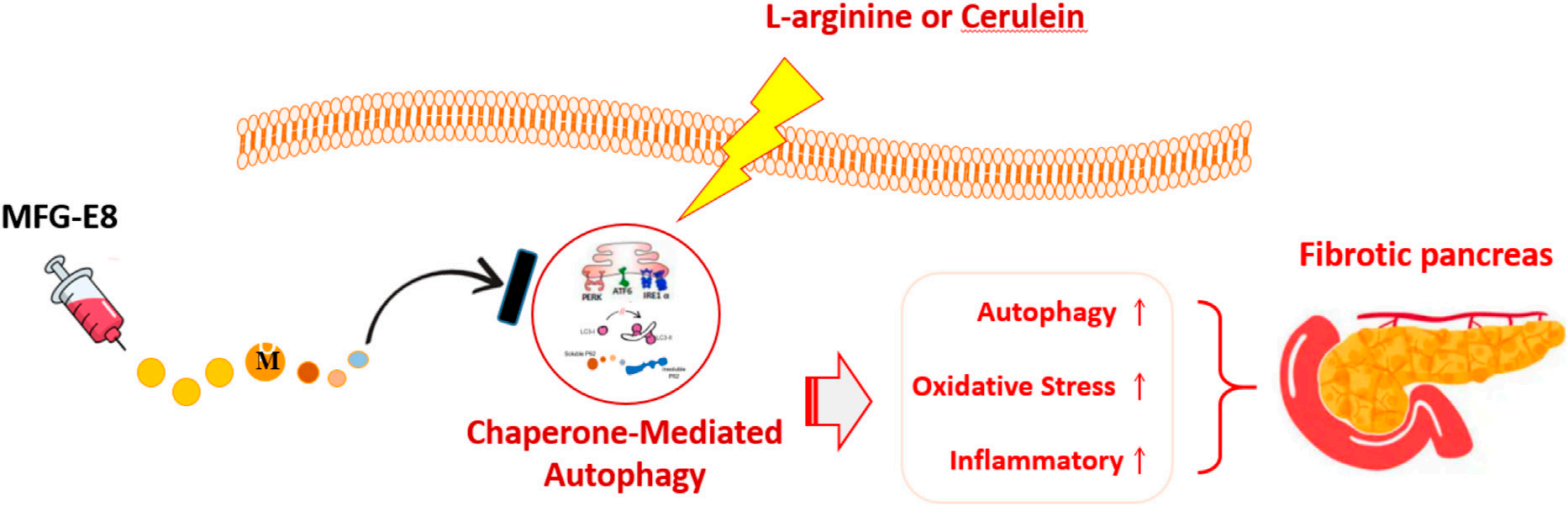
In this study, we found, for the first time, that MFG-E8 gene defect exaggerated pancreatic fibrosis after repeated cerulean injection in mice, and intraperitoneal injection of exogenous MFG-E8 alleviated pancreatic fibrosis in cerulein-CP mice, suggesting that MFG-E8 is an important regulator of pancreatic fibrosis in CP (Figure 6).

### MFG-E8 Levels Are Decreased in the Pancreas of CP Mice and Exogenous MFG-E8 Treatment Is Beneficial in Experimental CP

**FIGURE 2:**
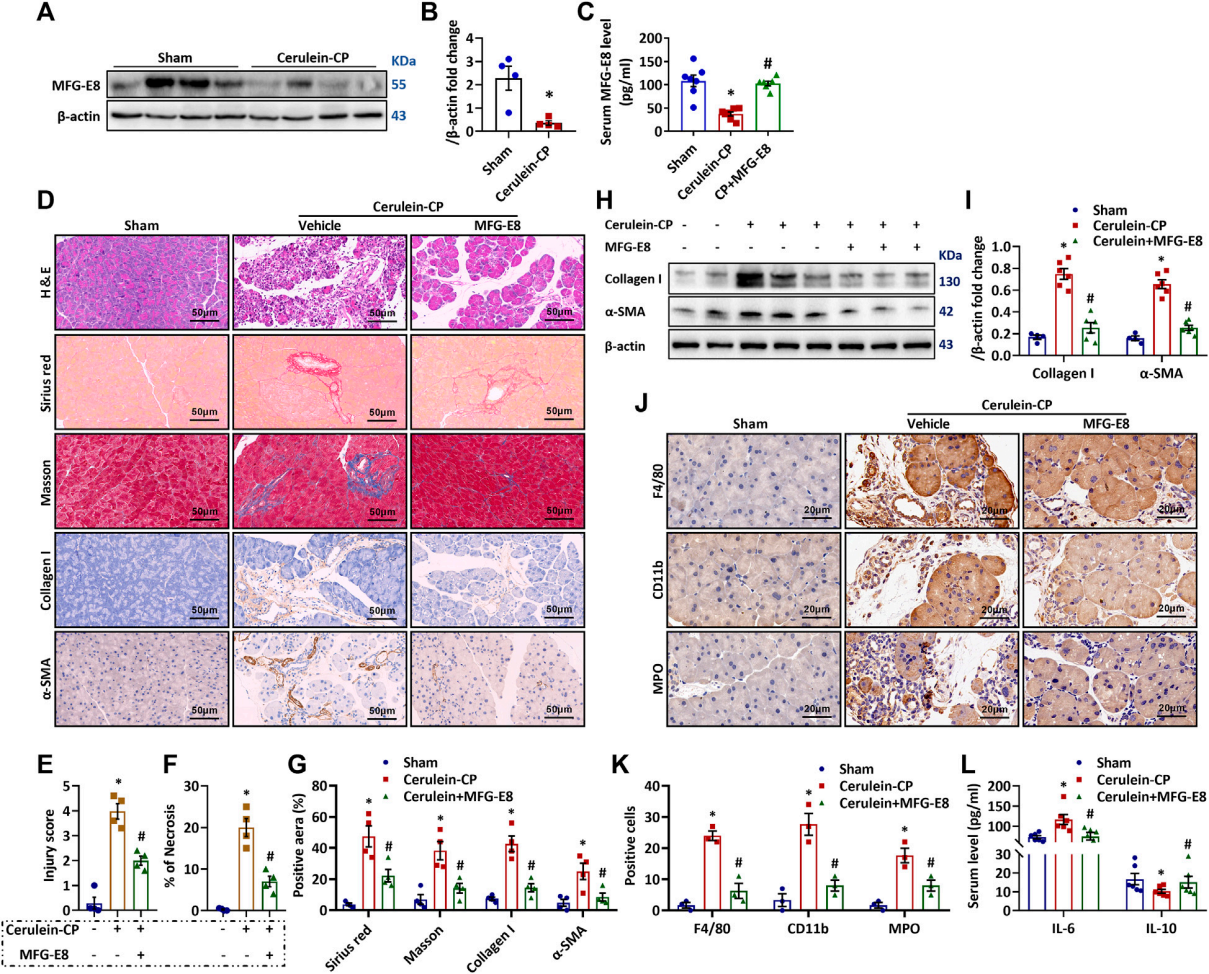
**Therapeutic Potential of Exogenous MFG-E8.** Cerulein-CP was induced by six IP injections of cerulein (50 μg/kg/body weight) twice a week for 10 weeks. During the last 5 weeks, 1 h after cerulein injection, normal saline (vehicle) or 20 μg/kg MFG-E8 was administered through intraperitoneal injection. The control group received the same frequency and time of intraperitoneal injection of normal saline (Sham). The animals were sacrificed at 2 days after the last injection of cerulein or normal saline. Blood and tissue samples were collected. **(A,B)** Western blot analysis of the expression of MFG-E8 in the pancreas (*t*-test); **(C)** Serum MFG-E8 levels (one-way ANOVA with the Tukey-Kramer test); **(D)** Representative photos of H&E, Sirius red, Masson, Collagen I and α-SMA staining; **(E)** Quantitative analysis of H&E staining (one-way ANOVA with the Tukey-Kramer test); **(F)** Percentages of necrotic areas (one-way ANOVA with the Tukey-Kramer test); **(G)** Quantitative analysis of Sirius red Masson, Collagen I and α-SMA staining (one-way ANOVA with the Tukey-Kramer test); **(H,I)** Western blot analysis of the expression of α-SMA and collagen I in the pancreas (one-way ANOVA with the Tukey-Kramer test); **(J)** Representative photos of F4/80, MPO and CD11b staining; **(K)** Quantitative analysis of F4/80, MPO and CD11b staining (one-way ANOVA with the Tukey-Kramer test); **(L)** Serum levels of IL-6 and IL-10 (one-way ANOVA with the Tukey-Kramer test). *n* = 4–6, mean ± SEM; ∗ *p* < 0.05 versus Sham group; #*p* < 0.05 versus Vehicle group. CP, chronic pancreatitis; MFG-E8, Milk Fat Globule-EGF Factor 8; H&E, hematoxylin and eosin; MPO, myeloperoxidase; α-SMA, alpha-smooth muscle actin.

To further investigate the role of MFG-E8 in CP, we measured MFG-E8 protein expression in the pancreas after repeated cerulein injection by western blot analysis. As shown in Figures 2A,B, repeated intraperitoneal injection of cerulein significantly decreased MFG-E8 protein levels in the pancreas of WT mice. Because MFG-E8 is a secreted protein, we also measured the serum MFG-E8 levels in mice. As shown in Figure 2C, serum MFG-E8 levels in cerulein-treated mice were significantly lower than those in the control group, while repeated intraperitoneal injection of exogenous MFG-E8 effectively maintained serum MFG-E8 levels (*p* < 0.05). Administration of recombinant MFG-E8 significantly reduced pancreatic injury (Figures 2D,E) and necrosis (Figure 2F) in cerulein-CP mice. Pancreatic fibrosis was evaluated by Sirius red, Masson, α-SMA and Collagen I staining. As shown in Figures 2D,G, exogenous MFG-E8 treatment reduced the positive staining of the above fibrosis-related indicators in the pancreatic tissue of cerulein-CP mice by 55.2, 59.5, 68.7, and 54.6%, respectively (*p* < 0.05). Western blot analysis also confirmed that α-SMA and Collagen I protein expression in pancreatic tissue of cerulein-CP mice was downregulated by MFG-E8 treatment (Figures 2H,I). Similar beneficial effects of exogenous MFG-E8 treatment was observed in repeated L-arginine injection-induced CP in mice (Supplementary Figures 2A–F). MFG-E8 also inhibited inflammatory responses in cerulein-CP mice. As shown in Figures 2J,K, administration of exogenous MFG-E8 reduced the numbers of F4/80, CD11b and MPO positive cells in the pancreas of cerulein-CP mice by 82.6, 72.5 and 47.9%, respectively (*p* < 0.05). The abnormal serum levels of inflammatory mediators (IL-6 and IL-10) in cerulein-CP mice were restored to almost sham levels by exogenous MFG-E8 treatment (Figure 2L).

### Exogenous MFG-E8 Treatment Alleviates Autophagy and Oxidative Stress in CP Mice

**FIGURE 3:**
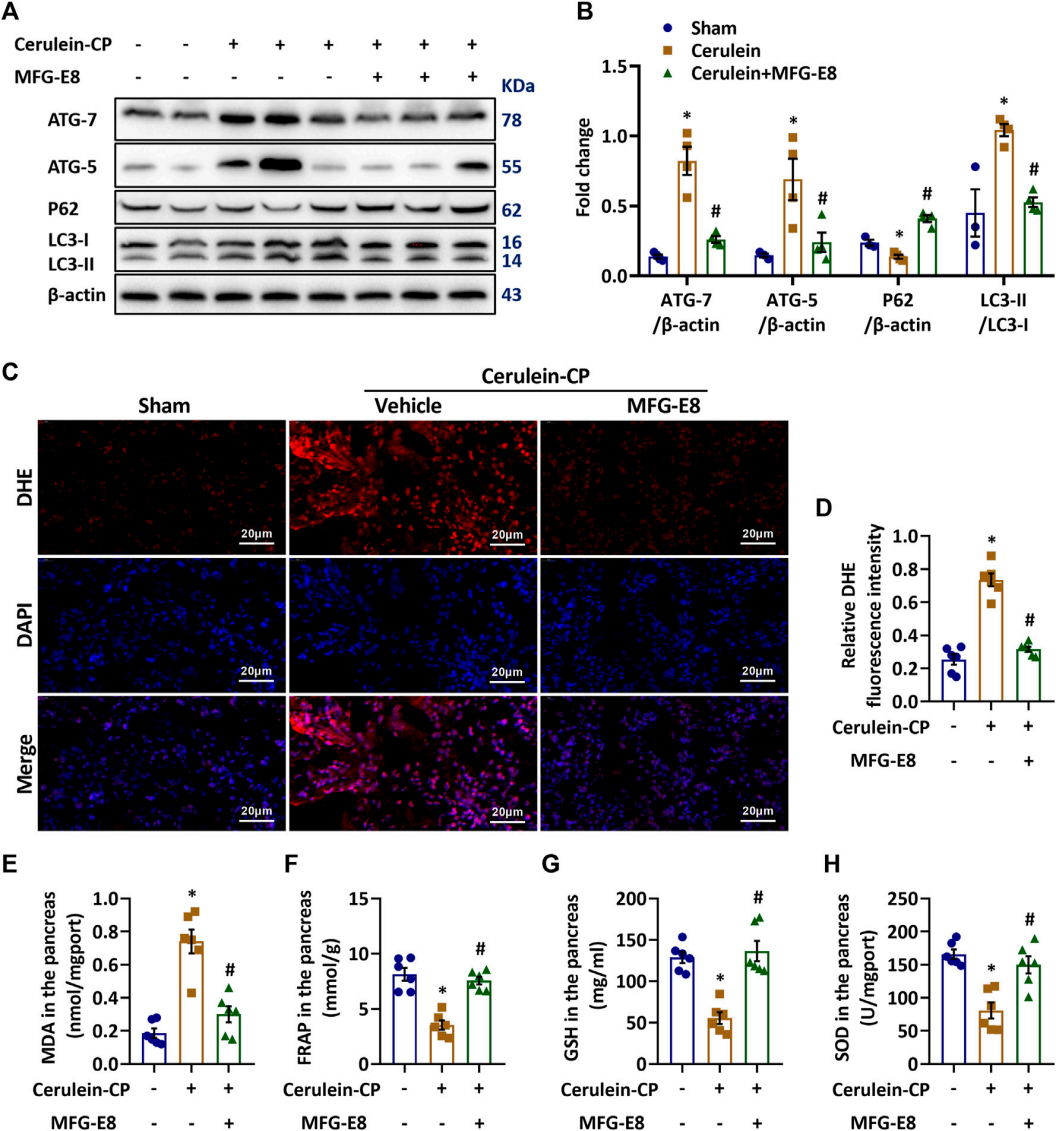
**Exogenous MFG-E8 Alleviates Autophagy and Oxidative Stress in Cerulein-CP.** Cerulein-CP was induced by six IP injections of cerulein (50 μg/kg/body weight) twice a week for 10 weeks. During the last 5 weeks, 1 h after cerulein injection, normal saline (vehicle) or 20 μg/kg MFG-E8 was administered through intraperitoneal injection. The control group received the same frequency and time of intraperitoneal injection of normal saline (Sham). The animals were sacrificed at 2 days after the last injection of cerulein or normal saline. Blood and tissue samples were collected. **(A,B)** Western blot analysis of the expression of ATG7, ATG5, P62 and LC3B in the pancreas (one-way ANOVA with the Tukey-Kramer test); **(C,D)** Representative images and quantitative analysis of immunofluorescence staining of DHE in the pancreas (one-way ANOVA with the Tukey-Kramer test); **(E)** FRAP level in the pancreas (one-way ANOVA with the Tukey-Kramer test); **(F)** GSH level in the pancreas (one-way ANOVA with the Tukey-Kramer test); **(G)** MDA level in the pancreas (one-way ANOVA with the Tukey-Kramer test); **(H)** SOD level in the pancreas (one-way ANOVA with the Tukey-Kramer test). *n* = 3–6, mean ± SEM; ∗ *p* < 0.05 versus Sham group; #*p* < 0.05 versus Vehicle group. CP, chronic pancreatitis; MFG-E8, Milk Fat Globule-EGF Factor 8; MDA, malondialdehyde; SOD, superoxide dismutase; FRAP, Ferric ion reducing antioxidant power; DHE, Dihydroethidium; GSH, glutathione.

Impaired autophagy and oxidative stress activate PSCs and promote their release of large amounts of extracellular matrix (ECM), which, along with collagen deposition, initiates and accelerates the progression of pancreatic fibrosis (Bhardwaj and Yadav, 2013; Ryu et al., 2013; Diakopoulos et al., 2015; Li et al., 2018a). To investigate the mechanism responsible for MFG-E8’s beneficial effects in CP, we measured indicators of autophagy and oxidative stress. We have confirmed that deleting the exons 4 to 6 of the MFG-E8 gene (*mfge8*-knockout) has no significant effect on the antioxidant capacity (Ren et al., 2021) and autophagy (Supplementary Figure S3) in the mouse pancreatic tissue. In this study, we further explored the effects of exogenous MFG-E8 on the levels of oxidative stress and autophagy in the pancreas of cerulein-treated CP mice. As shown in Figures 3A,B, pancreatic levels of ATG7, ATG5 and LC3 II/LC3 I increased, while P62 decreased significantly after repeated cerulein injection, indicating activated autophagy process in CP. Exogenous MFG-E8 treatment reversed these changes in cerulein-CP mice, suggesting MFG-E8 suppresses autophagy in CP. The enhancement of autophagy could induce the disorder of oxygen free radical regulation, resulting in oxidative stress (Li et al., 2021). Our results also indicated that repeated cerulein injection induced oxidative stress in the pancreas. As shown in Figures 3C,D, DHE staining in the pancreas increased dramatically after repeated cerulein injection. Consistently, pancreatic tissues levels of MDA (Figure 3E) were also significantly elevated in cerulein-CP. In the meantime, anti-oxidative indicators including FRAP (Figure 3F), GSH (Figure 3G) and SOD (Figure 3H) decreased after repeated cerulein injection. Exogenous MFG-E8 treatment decreased DHE, MDA, and increased FRAP, GSH, and SOD in the pancreas of cerulein-CP mice, suggesting MFG-E8 reduces oxidative stress in CP.

### MFG-E8 Blocks TGF-β1-Induced PSC Activation, Autophagy and Oxidative Stress *in Vitro*

**FIGURE 4:**
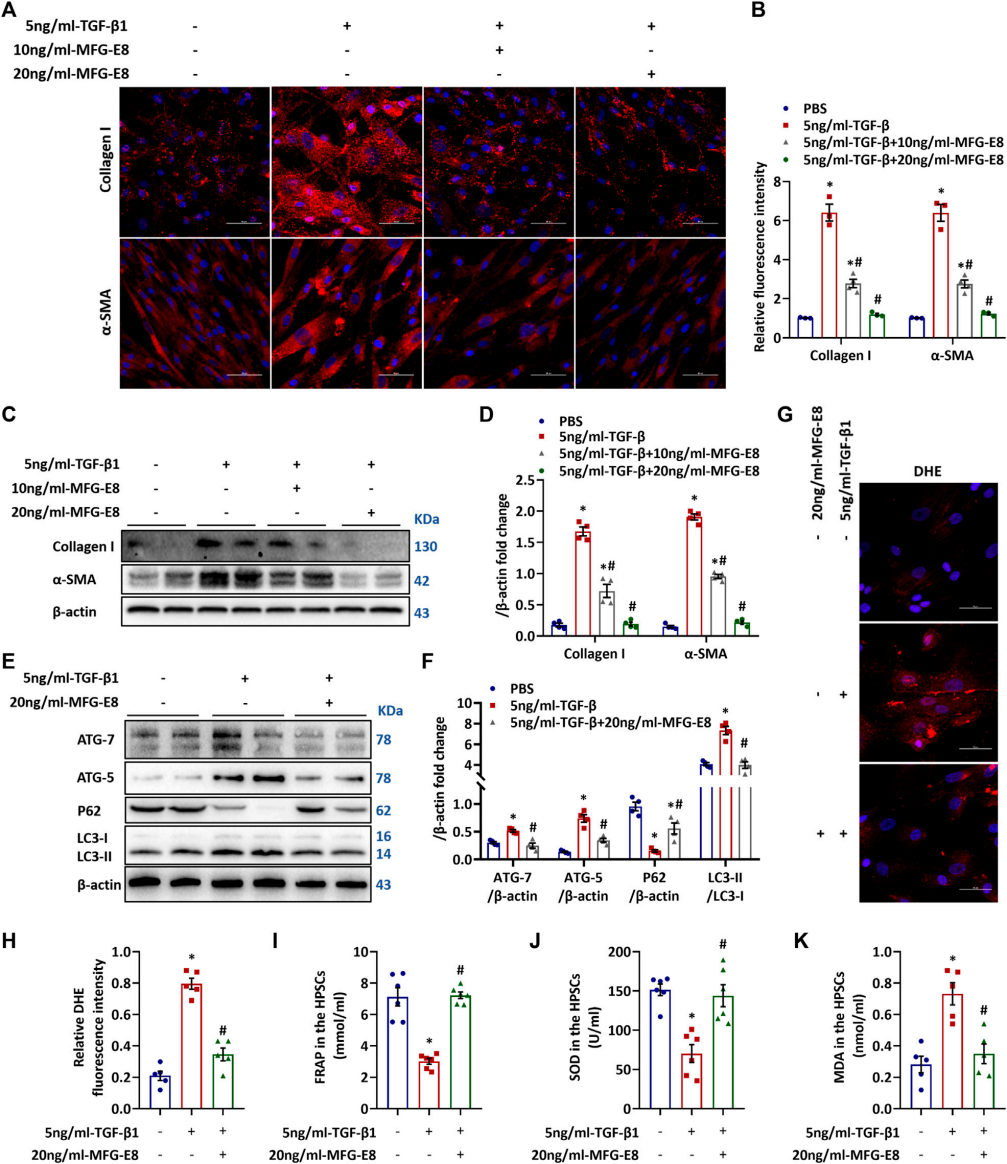
**Exogenous MFG-E8 Blocks TGF-β1–induced activation of HPSCs.** Human pancreatic stellate cells (1×10^6^/well) were treated with 5 ng/ml TGF-β1 with or without 10 ng/ml or 20 ng/ml MFG-E8 for 24 h, the same volume of PBS was added to another group of HPSCs as a control group. **(A,B)** Representative images and quantitative analysis of immunofluorescence staining of α-SMA and collagen I in the HPSCs (one-way ANOVA with the Tukey-Kramer test); **(C,D)** Western blot analysis of the expression of α-SMA and collagen I in the HPSCs (one-way ANOVA with the Tukey-Kramer test); **(E,F)** Western blot analysis of the expression of ATG7, ATG5, P62 and LC3B in the HPSCs (one-way ANOVA with the Tukey-Kramer test); **(G,H)** Representative images and quantitative analysis of immunofluorescence staining of DHE in the HPSCs (one-way ANOVA with the Tukey-Kramer test); **(I)** FRAP level in the HPSCs (one-way ANOVA with the Tukey-Kramer test); **(J)** SOD level in the HPSCs (one-way ANOVA with the Tukey-Kramer test); **(K)** MDA level in the HPSCs (one-way ANOVA with the Tukey-Kramer test). *n* = 3–6, mean ± SEM; ∗ *p* < 0.05 versus Sham group; #*p* < 0.05 versus Vehicle group. α-SMA, alpha-smooth muscle actin; TGF-β1, transforming growth factor-β1; HPSCs, human pancreatic stellate cells, MDA, malondialdehyde; SOD, superoxide dismutase; MFG-E8, Milk Fat Globule-EGF Factor 8; FRAP, Ferric ion reducing antioxidant power; DHE, Dihydroethidium.

PSCs activation plays a fundamental role in the development of pancreatic fibrosis. Activated PSCs have upregulated α-SMA expression and release a large amount of extracellular matrix proteins such as collagen I. To determine the effects of MFG-E8 on PSCs activation *in vitro*, we treated human PSCs with TGF-β1 in the presence of various concentrations of MFG-E8. As shown in Figures 4A–D, MFG-E8 dose-dependently suppressed TGF-β1-induced collagen I and α-SMA production in cultured human PSCs. MFG-E8 also blocked TGF-β1-induced autophagy (Figures 4E,F) and oxidative stress (Figures 4G–K) in cultured human PSCs.

### MFG-E8 Suppresses ER Stress and Chaperone-Mediated Autophagy in Activated PSCs

**FIGURE 5:**
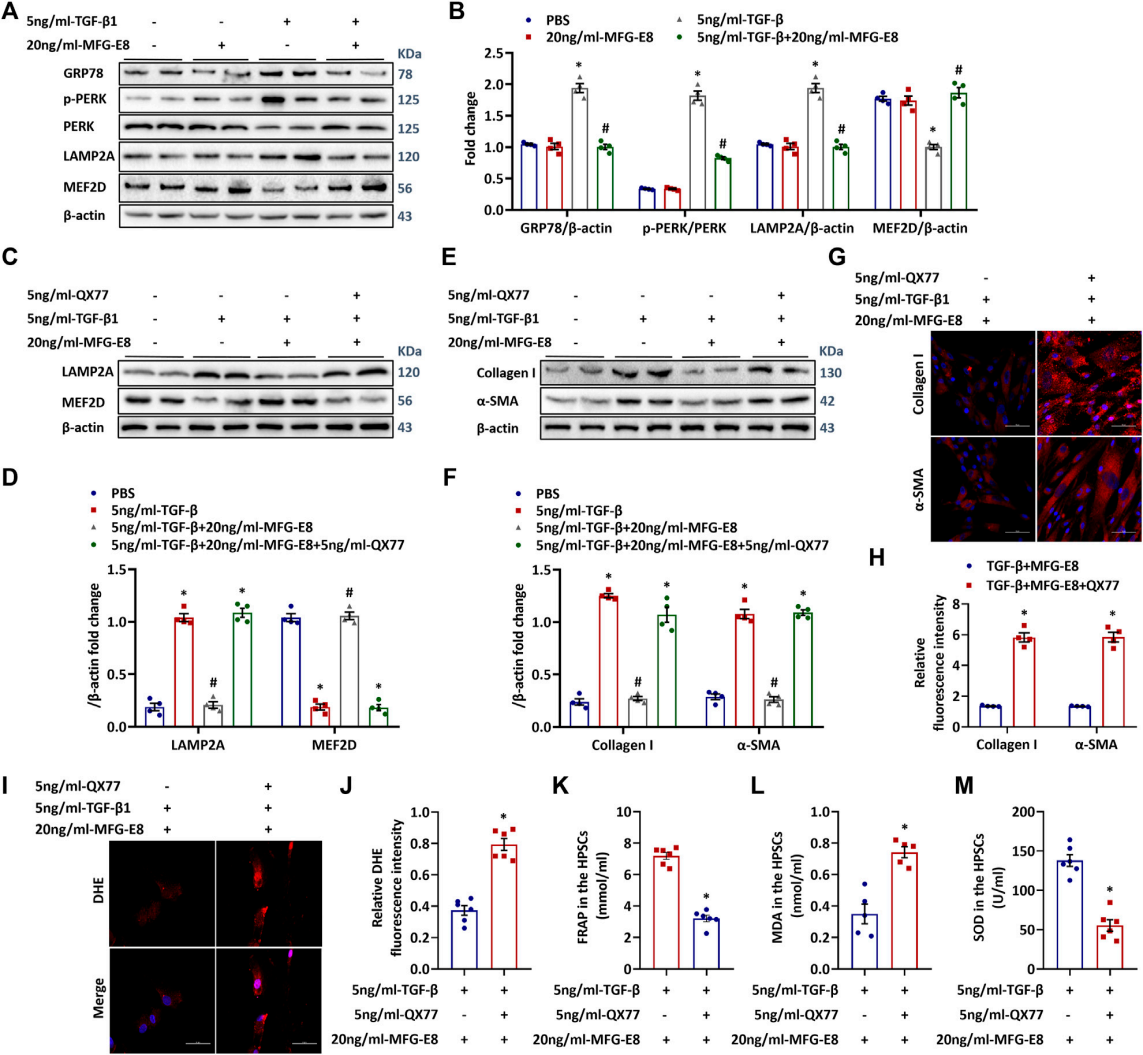
**ER Stress Induced CMA Mediate the Activation of HPSCs.** Human pancreatic stellate cells (1×10^6^/well) were treated with 5 ng/ml TGF-β1 with or without 20 ng/ml MFG-E8 for 24 h, the same volume of PBS was added to another group of HPSCs as a control group. To determine the role of ER stress-mediated CMA activation in exogenous MFG-E8’s effect in HPSCs, QX77, a LAMP2A-specific activator, was added simultaneously with 20 ng/mL-MFG-E8 in TGF-β1-treated HPSCs. **(A,B)** Western blot analysis of the expression of GRP78, p-PERK, PERK, LAMP2A and MEF2D in the HPSCs (one-way ANOVA with the Tukey-Kramer test); **(C,D)** Western blot analysis of the expression of LAMP2A and MEF2D in the HPSCs (one-way ANOVA with the Tukey-Kramer test); **(E,F)** Western blot analysis of the expression of α-SMA and Collagen I in the HPSCs (one-way ANOVA with the Tukey-Kramer test); **(G,H)** Representative images and quantitative analysis of immunofluorescence staining of α-SMA and collagen I in the HPSCs (*t*-test); **(I,J)** Representative images and quantitative analysis of immunofluorescence staining of DHE in the HPSCs (*t*-test); **(K)** FRAP level in the HPSCs (*t*-test); **(L)** MDA level in the HPSCs (*t*-test); **(M)** SOD level in the HPSCs (*t*-test). *n* = 4–6, mean ± SEM; ∗ *p* < 0.05 versus Sham group; #*p* < 0.05 versus Vehicle group. α-SMA, alpha-smooth muscle actin; TGF-β1, transforming growth factor-β1; HPSCs, human pancreatic stellate cells, MFG-E8, Milk Fat Globule-EGF Factor 8; DHE, Dihydroethidium; LAMP2A, Lysosomal associated membrane proteins 2a; MDA, malondialdehyde; SOD, superoxide dismutase; FRAP, Ferric ion reducing antioxidant power.

TGF-β1 treatment increased ER stress-related protein GRP78 expression and PERK phosphorylation in human PSCs (Figures 5A,B), suggesting activated ER stress. MFG-E8 decreased TGF-β1-induced GRP78 expression and PERK phosphorylation in human PSCs. ER stress can lead to CMA (Li et al., 2018b). LAMP2A is the rate-limiting receptor for CMA substrate flux. And increased CMA activity leads to MEF2D degradation (Li et al., 2017). As shown in Figures 5A,B, TGF-β1 also increased LAMP2A expression and decreased MEF2D expression in human PSCs, suggesting increased CMA activity. MFG-E8 decreased TGF-β1-induced LAMP2A express, while increased MEF2D expression in the meantime. Exogenous MFG-E8 also reduced ER stress and CMA in cerulein-treated CP mice (*p* < 0.05, Supplementary Figures S4A,B). QX77 is a specific CMA activator. It can upregulate LAMP2A expression (Zhang et al., 2017). As shown in Figures 5C,D, QX77 reversed MFG-E8’s effects on LAMP2A and MEF2D expression. To explore the role of CMA in MFG-E8’s effects on PSC activation, the expression of collagen I and α-SMA was measured. As shown in Figures 5E–H, QX77 eliminated MFG-E8’s effects on collagen I and α-SMA expression. Similarly, the suppressive effect of MFG-E8 on oxidative stress in activated PSCs was also mitigated by the addition of QX77 (Figures 5I–M).

## Discussion

Pancreatic fibrosis, a characteristic feature of CP, is the result of abnormal activation of stromal cells and deposition of extracellular matrix (ECM) proteins. The development of fibrosis leads to the gradual loss of exocrine and endocrine functions of the pancreas. Currently, there is no specific treatment for pancreatic fibrosis. Clinical management of CP patients mainly relies on supportive therapies to alleviate pain and prevent complications. As such, identifying key factors in pancreatic fibrosis would greatly contribute to the development of effective treatment for CP. In this study, we found, for the first time, that MFG-E8 gene defect exaggerated pancreatic fibrosis after repeated cerulein injection in mice, and intraperitoneal injection of exogenous MFG-E8 alleviated pancreatic fibrosis in cerulein-CP mice, suggesting that MFG-E8 is an important regulator of pancreatic fibrosis in CP.

MFG-E8 was first identified in the lactation mammary gland (Hanayama and Nagata, 2005). Subsequent studies have demonstrated that MFG-E8 promotes the removal of apoptotic cells and inhibits inflammatory responses (Miksa et al., 2008; Kranich et al., 2010; Wu et al., 2010; Cheyuo et al., 2012; Shah et al., 2012). MFG-E8 deficiency has been linked to the development of autoimmune diseases such as rheumatoid arthritis and inflammatory bowel disease (Nagata, 2007; Albus et al., 2016; He et al., 2016). Our recent study has shown that MFG-E8 restores mitochondrial function *via* integrin-medicated activation of the FAK-STAT3 signaling pathway in acute pancreatitis (Ren et al., 2021). It is well known that repeated episodes of acute pancreatitis lead to the development of CP. To extend our investigation of MFG-E8 in pancreatitis, we evaluated the role of MFG-E8 in pancreatic fibrosis in the current study. The results suggest that MFG-E8 has an anti-fibrotic property in the pancreas. This is consistent with the reported function of MFG-E8 in hepatic fibrosis, renal fibrosis and skin fibrosis (Fujiwara et al., 2019; Shi et al., 2020; Wang et al., 2020). Thus, MFG-E8 may be a promising option for the treatment of pancreatic fibrosis.

Activation of PSCs plays a critical role in the development of pancreatic fibrosis in CP (Ramakrishnan et al., 2020). Activated stellate cells cause the deposition of extracellular matrix by releasing a series of collagen fibers including α-SMA, collagen I and III. Pathological changes were manifested as the loss of a large number of functional cells such as pancreatic acinus cells and pancreatic-beta cells, and replaced by a large number of proliferation of non-functional extracellular matrix (Masamune et al., 2009). Autophagy is necessary for the activation of PSCs (Endo et al., 2017). The increase of intracellular oxygen free radicals induced by autophagy aggravates oxidative stress and further stimulates the release of α-SMA by activated PSCs (Xue et al., 2019). Using two different mouse models of CP, we showed that intraperitoneal injection of exogenous MFG-E8 inhibited pancreatic fibrosis and inflammatory responses. Moreover, in our *in vitro* study, we found that exogenous MFG-E8 alleviated TGF-β1-induced activation of human PSCs, which is associated with reduced autophagy and oxidative stress, indicating that MFG-E8 might inhibit the activation of PSCs by suppressing autophagy.

Our previous study has found that TGF-β1-induced activation of HPSCs results in ER stress and aggravates cellular oxidative stress (Ren et al., 2020). ER stress leads to the activation of chaperone-mediated autophagy (CMA) (Abokyi et al., 2020). CMA is a unique form of autophagy, which was only found in mammalian cells. It requires the participation of lysosomal-associated membrane protein 2A (LAMP2A), which facilitates the translocation of cytosolic proteins containing a KFERQ-like peptide motif across the lysosomal membrane and subsequent MEF2D degradation (Pajares et al., 2018; Wang et al., 2018). As a rate-limiting molecule of CMA, the abnormal expression or function of LAMP2A is of great pathophysiological significance. In the current study, we found that TGF-β1 treatment led to the elevated expression of LAMP2A and the reduced level of MEF2D. MFG-E8, on the other hand, decreased LAMP2A expression and increased MEF2D expression in TGF-β1-treat human PSCs, suggesting MFG-E8 can suppress the CMA pathway. QX77, a specific CMA activator, not only reversed MFG-E8’s effects on LAMP2A and MEF2D expression, but also eliminated MFG-E8’s effects on collagen I and α-SMA expression. These results, taken together, indicated that MFG-E8 mitigates pancreatic fibrosis by inhibiting the ER stress-induced CMA pathway. However, it is also possible that MFG-E8 directly inhibits ER stress. A recent study by Song M et al. has shown that activation of p-STAT3 alleviates ER-stress in splenocytes during chronic stress (Song et al., 2020). Our previous study has suggested that MFG-E8 can activate p-STAT3 (Ren et al., 2021). In this regard, the direct effect of MFG-E8 on ER-stress in pancreatitis warrants further investigation.

The impact of MFG-E8 in CP, however, remains controversial. D'Haese JG et al. found that compared with normal pancreatic tissue samples obtained from healthy organ donors, pancreatic tissues collected from chronic pancreatitis patients had significantly higher levels of MFG-E8 (D’Haese et al., 2013). How the normal pancreatic tissue samples were obtained and preserved, however, were not described in the paper. Pancreatic tissues obtained from organ donors might undergo ischemia reperfusion injury, machine perfusion and static cold storage. All these factors could alter the expression of MFG-E8. More importantly, the authors did not provide any direct evidence showing MFG-E8 plays a pathogenic role in chronic pancreatitis. In the current study, we found that administration of recombinant MFG-E8 alleviated pancreatic fibrosis in mouse models of CP and MFG-E8 inhibited TGF-β1-induced ER stress and chaperone-mediated autophagy in cultured human PSCs. In addition, knockout of *mfge8* gene exaggerated pancreatic fibrosis after repeated cerulein injection in mice. These results were consistent with several other studies, which also showed that MFG-E8 has anti-fibrotic effects (Brissette et al., 2016; Fujiwara et al., 2019; Shi et al., 2020; Kim et al., 2021).

There are some limitations of the study. First of all, due to the lack of clinical samples, we were unable to verify our findings in CP patients. The clinical significance of this study warrants further investigation. And alcohol consumption is the most common cause of CP in Western societies (Singh et al., 2019). Although we evaluated the anti-fibrotic effect of MFG-E8 in two different CP models, whether it has any effect on alcohol-induced CP remains unknown. The major biological effects of MFG-E8 are mediated through binding to αvβ3/5 integrins. Our previous study has shown that administration of cilengitide, a specific αvβ3/5 integrin inhibitor, abolished MFG-E8’s beneficial effects in acute pancreatitis (Ren et al., 2021). Whether the recombinant MFG-E8 has any off-target effects in CP, however, remains to be determined. Furthermore, this study showed that MFG-E8 downregulated LAMP2A expression. However, the detailed molecular mechanism is still unknown.

## Conclusion

MFG-E8 alleviates pancreatic fibrosis *via* inhibiting ER stress-induced chaperone-mediated autophagy in experimental CP. Recombinant MFG-E8 may be developed as a novel treatment for pancreatic fibrosis in CP.

## Data Availability

The raw data supporting the conclusion of this article will be made available by the authors, without undue reservation.
