# Supplementary material for: Milk Fat Globule-EGF Factor 8 Alleviates Pancreatic Fibrosis by Inhibiting ER Stress-Induced Chaperone-Mediated Autophagy in Mice
Source: Front Pharmacol. 2021 Aug 5;12:707259. doi: 10.3389/fphar.2021.707259 (PMC8375434; doi:10.3389/fphar.2021.707259)
Supplement: Supplementary file 1 [file DataSheet1.docx]

Supplementary Materials for

**Milk Fat Globule-EGF Factor 8 Alleviates Pancreatic Fibrosis by Inhibiting ER Stress-Induced**

**Chaperone-Mediated Autophagy in Mice**

Yifan Ren, Qing Cui, Jia Zhang, Wuming Liu, Meng Xu, Yi Lv, Zheng Wu, Yuanyuan Zhang, Rongqian Wu

*Corresponding author: [rwu001@mail.xjtu.edu.cn](mailto:rwu001@mail.xjtu.edu.cn)

**This PDF file includes:**

Supplementary Table 1

Supplementary Figure 1

Supplementary Figure 2

Supplementary Figure 3

Supplementary Figure 4

**Supplementary Table 1: Antibodies**

| **Antibody** | **Item No** | **Company and location** |
| --- | --- | --- |
| α-SMA Rabbit mAb | 19245 | Cell Signaling Technology, Beverly, MA, USA |
| Anti-Collagen I antibody | ab34710 | Abcam, Cambridge, MA, USA |
| ATG7 (D12B11) Rabbit mAb | 8558 | Cell Signaling Technology, Beverly, MA, USA |
| Anti-β-actin | 60008 | proteintech, CN |
| ATG5 (D5F5U) Rabbit mAb | 12994 | Cell Signaling Technology, Beverly, MA, USA |
| Phospho-SQSTM1/p62 Rabbit mAb | 16177 | Cell Signaling Technology, Beverly, MA, USA |
| Anti-LAMP2A | ab125068 | Abcam, Cambridge, MA, USA |
| LC3B Antibody | 2775 | Cell Signaling Technology, Beverly, MA, USA |
| BiP (C50B12) Rabbit mAb | 3177 | Cell Signaling Technology, Beverly, MA, USA |
| MFG-E8 antibody | sc-271574 | SANTA CRUZ Biotechnology, Texas, USA |
| MEF2D (E9R5J) Rabbit mAb | 25651 | Cell Signaling Technology, Beverly, MA, USA |
| PERK (C33E10) Rabbit mAb | 3192 | Cell Signaling Technology, Beverly, MA, USA |
| Phospho-PERK (Thr982) Antibody | DF7576 | Affinity Biosciences, CN |
| Goat anti-Mouse IgG antibody | 31430 | PIONEER Biotechnology, CN |
| Goat anti-Rabbit IgG antibody | 31460 | PIONEER Biotechnology, CN |

**
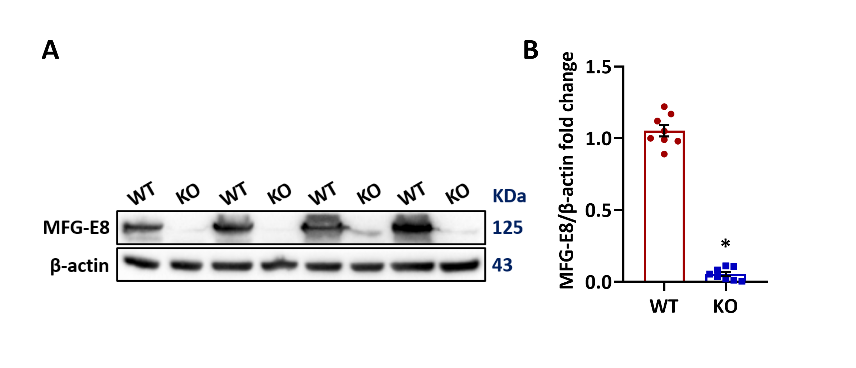
**

**Supplementary Figure 1. Western blot analysis of MFG-E8 in the pancreatic tissue of WT and *mfge8* KO mice.** (**A**) Representative blots; (**B**) Quantitative analysis (t-test). n = 8, mean ± SEM; ∗ P<0.05 versus WT group. MFG-E8, Milk Fat Globule-EGF Factor 8; WT, wild type; KO, knockout.

**
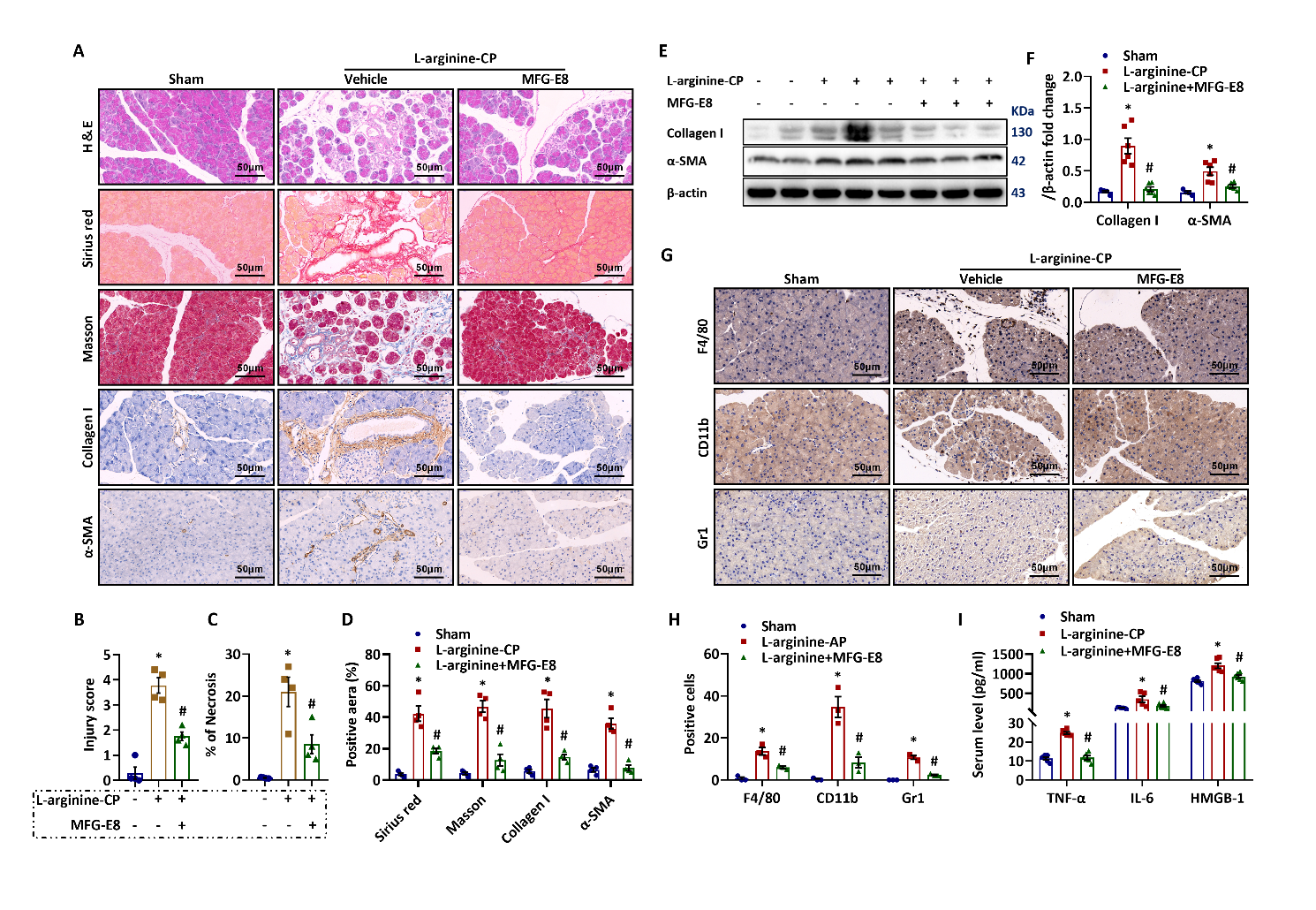
**

**Supplementary Figure 2. Exogenous MFG-E8 alleviates L-arginine-induced CP in mice.** L-arginine-CP was induced by 2 hourly intraperitoneal injections of 4.0 g/kg l-arginine twice a week for 10 weeks. Two hours after the last injection of L-arginine, normal saline (vehicle) or 20 μg/kg MFG-E8 were administered intraperitoneally. The control group received the same frequency and time of intraperitoneal injection of normal saline (Sham). The animals were sacrificed at 2 days after the last injection of L-arginine or normal saline. Blood and tissue samples were collected. (**A**) Representative photos of H&E, Sirius red, Masson, Collagen I and α-SMA staining; (**B**) Quantitative analysis of H&E staining (one-way ANOVA with the Tukey-Kramer test); (**C**) Percentages of necrotic areas (one-way ANOVA with the Tukey-Kramer test); (**D**) Quantitative analysis of Sirius red Masson, Collagen I and α-SMA staining (one -way ANOVA with the Tukey-Kramer test); (**E&F**) Western blot analysis of the expression of α-SMA and collagen I in the pancreas (one -way ANOVA with the Tukey-Kramer test); (**G**) Representative photos of F4/80, Gr1 and CD11b staining; (**H**) Quantitative analysis of F4/80, Gr1 and CD11b staining (one -way ANOVA with the Tukey-Kramer test); (**I**) Serum levels of TNF-α, IL-6 and HMGB-1 (one -way ANOVA with the Tukey-Kramer test). n = 4-6, mean ± SEM; ∗ P<0.05 versus Sham group; # P<0.05 versus Vehicle group. CP, chronic pancreatitis; MFG-E8, Milk Fat Globule-EGF Factor 8; H&E, hematoxylin and eosin; α-SMA, alpha-smooth muscle actin.


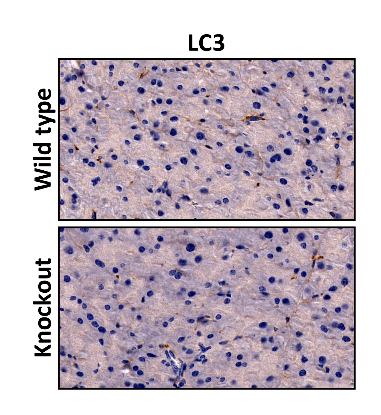


**Supplementary Figure 3. Knockout of mfge8 gene in mice did not affect pancreatic autophagy.** Representative photos of LC3B staining.


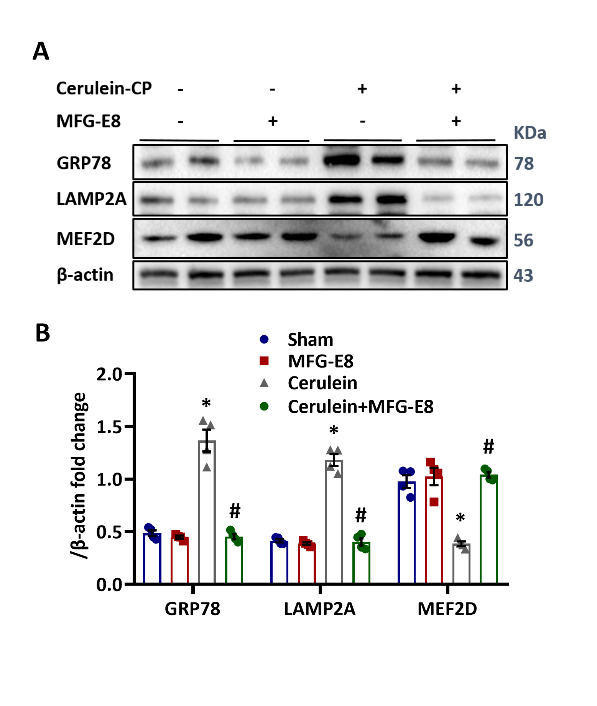


**Supplementary Figure 4. Exogenous MFG-E8 alleviates ER stress-induced CMA in cerulein-treated CP mice.** Cerulein-CP was induced by 6 IP injections of cerulein (50 μg/kg/body weight) twice a week for 10 weeks. During the last five weeks, one hour after cerulein injection, normal saline (vehicle) or 20 μg/kg MFG-E8 was administered through intraperitoneal injection. The control group received the same frequency and time of intraperitoneal injection of normal saline (Sham). The animals were sacrificed at 2 days after the last injection of cerulein or normal saline. Blood and tissue samples were collected. (**A&B**) Western blot analysis of the expression of GRP78, LAMP2A and MEF2D in the pancreas (one-way ANOVA with the Tukey-Kramer test). n = 4, mean ± SEM; ∗ P<0.05 versus Sham group; # P<0.05 versus Vehicle group. CP, chronic pancreatitis; MFG-E8, Milk Fat Globule-EGF Factor 8; LAMP2A, Lysosomal associated membrane proteins 2a.
